# Using a novel climate–water conflict vulnerability index to capture double exposures in Lake Chad

**DOI:** 10.1007/s10113-016-1003-6

**Published:** 2016-07-06

**Authors:** Uche T. Okpara, Lindsay C. Stringer, Andrew J. Dougill

**Affiliations:** Sustainability Research Institute, School of Earth and Environment, Faculty of Environment, University of Leeds, Leeds, LS2 9JT United Kingdom

**Keywords:** Double exposure, Climate variability, Water conflict, Vulnerability assessment, Human security

## Abstract

Climate variability is amongst an array of threats facing agricultural livelihoods, with its effects unevenly distributed. With resource conflict being increasingly recognised as one significant outcome of climate variability and change, understanding the underlying drivers that shape differential vulnerabilities in areas that are double-exposed to climate and conflict has great significance. Climate change vulnerability frameworks are rarely applied in water conflict research. This article presents a composite climate–water conflict vulnerability index based on a double exposure framework developed from advances in vulnerability and livelihood assessments. We apply the index to assess how the determinants of vulnerability can be useful in understanding climate variability and water conflict interactions and to establish how knowledge of the climate–conflict linked context can shape interventions to reduce vulnerability. We surveyed 240 resource users (farmers, fishermen and pastoralists) in seven villages on the south-eastern shores of Lake Chad in the Republic of Chad to collect data on a range of exposure, sensitivity and adaptive capacity variables. Results suggest that pastoralists are more vulnerable in terms of climate-structured aggressive behaviour within a lake-based livelihoods context where all resource user groups show similar levels of exposure to climate variability. Our approach can be used to understand the human and environmental security components of vulnerability to climate change and to explore ways in which conflict-structured climate adaptation and climate-sensitive conflict management strategies can be integrated to reduce the vulnerability of populations in high-risk, conflict-prone environments.

**Electronic supplementary material:**

The online version of this article (doi:10.1007/s10113-016-1003-6) contains supplementary material, which is available to authorized users.

## Introduction

The growing effects of climate variability and change have triggered an array of vulnerability assessment models seeking to identify ways to protect vulnerable people and livelihoods in locations exposed to perturbations and stresses (e.g. Antwi-Agyei et al. 2012; Reed et al. 2013; Wiréhn et al. 2015). Livelihood research recognises the need to understand how human and environmental conditions influence the means to make a living (Scoones 2009). This understanding often finds relevance in place-based livelihood vulnerability analysis, where methods to operationalise vulnerability focus on the specificity of localised concerns or variables (Turner et al. 2003). The concept of double exposure was popularised by O’Brien and Leichenko (2000) in their accounts of climate change and economic globalisation interactions. The concept invokes the notion of multiple and overlapping processes of change (biophysical and socio-economic) that take place within particular localities. It emphasises how contextual conditions influence the exposure and capacities of populations to create new contexts for experiencing and responding to change (Silva et al. 2010). This perspective has been applied to understand vulnerability through the lens of conflict-generating social dynamics across national and sub-national scales (Mason et al. 2011; Busby et al. 2013; Ide et al. 2014).

The climate security research community now recognises that exposure to climate and conflict stresses presents a critical challenge for locations where natural resources are declining and livelihood losses are driving people into conflict-structured practices (Gemenne et al. 2014; Scheffran et al. 2014). Discourses in this domain are useful for stressing human security and climate vulnerability concerns (Detraz 2011). Nonetheless, research into what makes a place vulnerable to the conflict consequences of climate change has tended to create more confusion than answers (Buhaug 2015). This may be related to a limited strategic understanding of ‘the nature of the state’ as against ‘the state of nature’ (see Raleigh et al. 2014), leaving a major gap in the literature where ‘views from the vulnerable’ (Tschakert 2007) provide useful insight. Except for Busby et al.’s (2014) work on climate security vulnerability which maps ‘double-exposed’ locations in Africa using sub-national level data, vulnerability frameworks are rarely applied in climate conflict studies at household and community levels—which is the scale at which processes generating vulnerability can be narrowly defined and validated (Barnett et al. 2008). A bottom-up, systematic approach to operationalise climate–water conflict vulnerability holds promise in terms of teasing out the repertoire of interacting variables that influence climate and conflict relationships.

In this paper, we develop and apply a composite climate–water conflict vulnerability index (CWCVI) to: (1) identify and compare the vulnerabilities of farming, fishing and pastoral livelihoods in the Lake Chad basin to climate variability and water conflict; (2) assess whether and how the determinants of vulnerability can be useful in understanding climate and water conflict interactions; and (3) explore how a climate–conflict linked context in which vulnerability is experienced can inform interventions to reduce vulnerability in conflict-prone environments. Further, we introduce a double exposure index (DEI) as an embedded component of the CWCVI to capture differential ‘climate–water conflict’ exposures amongst different livelihood groups. The paper serves the ‘ground-truthing’ requirements for studies on climate change and conflict hotspot mapping (e.g. Busby et al. 2014; de Sherbinin 2014) where field-based data validation is essential. Our systematic, multi-method approach provides a methodological contribution in line with the demand to combine a diversity of approaches and methods to investigate the full complexity of climate conflict links in human–environment systems (Gemenne et al. 2014). Our focus on a village-level assessment in Lake Chad contributes to a growing strand of vulnerability literature which seeks to enhance the rigour and utility of indicator-based vulnerability assessments.

## Theoretical background

### Indicator-based vulnerability assessment

Vulnerability is perceived as a state of defencelessness or powerlessness for people threatened directly or indirectly by changing conditions caused by a single or a collection of stressors (O’Brien et al. 2009). In climate change impact studies, vulnerability often draws attention to notions of exposure, sensitivity and adaptive capacity (IPCC 2007), including fragility and human security (Barnett and Adger 2007). Vulnerability assessments focus on identifying determinants of vulnerability by investigating reasons behind unequal exposure, impacts or responses. The priority areas are usually to identify vulnerable places, people and sectors, to raise awareness on where adaptation funds should be directed and to monitor adaptation policies (Luers et al. 2003). These are recognised as necessary for enhancing the utility of vulnerability reduction strategies in development planning (Oppio et al. 2015).

Vulnerabilities of individuals and communities are initiated by different interacting biophysical and socio-economic stressors. The extent to which populations are able to protect themselves is contingent upon how they are able to adjust (Reid and Vogel 2006; Tschakert 2007). Yet, as O’Brien et al. (2004, 2009) point out, assessments are often undertaken in isolation from ongoing global negative interacting outcomes. This is often the case for social stressors driven by human conflict *(*Mason et al. 2011). However, vulnerability indicators are now widely applied to account for interacting shocks and stressors and in particular to enhance the communicative power of vulnerability assessment findings (Tonmoy et al. 2014). Indeed, growing interest in understanding the forces that shape the state of affairs in vulnerable countries has made the use of vulnerability indicators relevant in vulnerability hotspot mapping (Hinkel 2011; Abson et al. 2012).

Several criticisms have been raised regarding the scientific novelty and policy relevance of vulnerability indicators. Many suggest that indicators are ‘a typical example of failed science-policy communication’ (Hinkel 2011: p. 199) particularly in relation to the non-transparent manner in which methodologies for developing indicators are presented (Eriksen and Kelly 2006; Barnett et al. 2008). Scientific definitions and frameworks guiding vulnerability assessments are generally imprecise about methodologies. For example, Working Group II of the International Panel on Climate Change (IPCC 2007) identifies exposure, sensitivity and adaptive capacity as the defining components of vulnerability. Yet the largely subjective connotation of these components makes it unclear how they can combine to capture vulnerability, as well as the relationships between them (Wolf et al. 2013). Many vulnerability indicators capture these components separately, paying limited or no attention to how to integrate them. The lack of communality in definitions has often meant that indicators must come from the specific research or policy questions considered (Wolf et al. 2013). Further, due to the place-based and context-specific nature of vulnerability, normative value judgement has tended to guide many vulnerability assessment methodologies (Hahn et al. 2009; Shah et al. 2013).

Despite criticisms concerning the use of indicators, their local relevance is widely supported (Barnett et al. 2008; Orencio and Fujii 2013). In this paper, we conceptualise vulnerability as a theoretical, non-observable phenomenon that relates to the propensity of a system, subsystem or system component to experience harm due to exposure to a perturbation or stressor (Turner et al. 2003). We apply observable variables or indicators to operationalise vulnerability, focusing on agricultural livelihoods in a lake-dependent environment.

### Framing climate conflict vulnerability in water-limited environments

Climate and conflict are rarely examined together in vulnerability science or within a single vulnerability framework (Eriksen and Lind 2005). Similarly, little work has advanced vulnerability models to capture climate and conflict stressors at household and community levels in locations facing severe water scarcity. The absence of a common narrative that explains vulnerability evidently influences how vulnerability to the water conflict consequences of climate change is understood and interpreted. Existing theoretical state-of-the-art literature seeking explanations for climate conflict highlights many methodological postulations which have produced more divisions than agreements (Buhaug 2015). The diversity of ways in which conflict is conceived is accompanied by a similar diversity of proxies used to quantify climate change across systems and within temporal and spatial scales. Conversations in this field typically draw upon the environmental security thesis (Homer-Dixon 1999; Le Billon 2001) as the basis for a theoretical understanding of the role of environmental resources in conflict events. Water has remained a key element in the literature given its characteristic feature as a resource worth fighting for (Cook and Bakker 2012), e.g. when power relations and ineffective water governance affect water sharing, particularly where rivers flow across state boundaries (Ludwig et al. 2011).

Although there is a rich literature on whether climate change impacts on water supplies is a factor in domestic conflicts, little is known about where the livelihood vulnerability literature fits in the environmental security discourse. The concept of vulnerability is less evident in water conflict studies compared with poverty, food security and disaster risk management studies. Mainstream writings (e.g. Böhmelt et al. 2014; Selby and Hoffmann 2014) explore indicators that suggest a pathway linking climate change and water conflict. Yet the literature remains vague regarding how vulnerability analysis may enable identification of interacting variables that shape both the demand for and supply of water, including efforts to restrain water conflict in lakeside villages where climate extremes are a major threat. To anticipate appropriate solutions for resource-dependent societies marred by conflict requires knowledge from the broad fields of climate security, livelihoods and vulnerability science to investigate the structures and processes that shape the propensity for livelihoods to be weakened by exposure to climate stressors and violence (Mason et al. 2011). Important aspects include, for example, knowing how people’s adaptability is shaped by socio-demographic profiles, livelihood strategies and social/political networks. Giving climate vulnerability a security focus (Scheffran et al. 2012) and knowing the vulnerability condition in which households and groups are ‘powerless’ or ‘wounded’ has huge practical significance (Füssel and Klein 2006).

## Study area and methodology

### Study area

Lake Chad’s water resources support agricultural livelihoods spanning rural villages in four countries (Cameroon, Chad, Niger and Nigeria) (Odada et al. 2006). Although the Lake lost more than 90 % of its waters between 1963 and 2012 (Lemoalle et al. 2012), the Chadian shore continued to hold a relatively large portion of the Lake’s remaining open waters, creating spaces for frequent trading and interactions amongst migrants of diverse ethnic groups. Our study focuses on the south-eastern shore and islands of the Lake Chad basin (12^o^53^″^N; 14^o^37^″^E), in the Haraze Al Biar administrative unit of the Republic of Chad. This location has a population of 1,50, 070 (Geohive 2015), characterised by villages that are geographically and politically remote.

Lake Chad is recognised as a location where human security is and will be progressively threatened as climate changes (Kafumbata et al. 2014). Thus, the precarious security situation, as evident in the manner the Lake environment acts as a cover for criminal and terrorist activities (Ifabiyi 2013), limited our choice of study location to seven villages^1^ in close proximity to the Lake. These were jointly selected for data collection in 2014 by the Lake Chad Basin Commission and a local NGO, the Chadian Indigenous Peule Mbororo Association. They are considered representative of farming, fishing and pastoral villages made up of livelihood groups that are generally and historically exposed to disruptive climate extremes and conflicts in the region (Table 1). Average annual rainfall is approximately 200–500 mm with maximum rainfall observed during July–September, while average temperature is approximately 27 °C, ranging from 21 to 36 °C throughout the year (Amaral et al. 2013). Since the 1970 s, intense droughts have impacted water supplies and, in turn, have intensified aggression and conflicts around the Lake for which several hundreds of lives have been lost (Onuoha 2009).

**Table 1:** A synthesis of climate and conflict events in Lake Chad based on secondary data sources

Exposure	Period	Source
Climate exposure		
Past droughts: four severe drought events recorded since 1970	1972–1975, 1982–1985, 1989–1992, 2002–2005	UNEP (2004, 2006)
Variation in past maximum temperature (^o^C) Reference period average: 36 Average of anomalies: 0.004 Standard deviation: 0.64	1960–2008	Computed from DREM (2013)
Variation in past minimum temperature (^o^C) Reference period average: 21 Average of anomalies: 0.07 Standard deviation: 0.81	1960–2008	DREM (2013)
Variation in past rainfall (mm) Long-term average: 436 Standard deviation: 111.79	1980–2008	DREM (2013)
Conflict exposure		
Boko Haram related (selected examples)		
Battle along the Chad/Nigeria border of Lake Chad in Kukawa killed hundreds of locals	19–20/4/2013	ACLED (2015)
Boko Haram killed 7 fishermen, injured 15 others, burnt boats and nets used for fishing on Lake Chad near Baga	28/11/2013	ACLED (2015)
Gunmen attacked a Lake Chad community (Malamfatori, Abadam LGA) in Chad killing 10	17/10/2014	ACLED (2015)
Fish traders ambushed, had their throats slit and drowned in Lake Chad (48 killed)	24/11/2014	ACLED (2015)
Three islands in Lake Chad attacked by gunmen, 19 local farmers and fishermen died of bullet wounds, fire and drowning	1/3/2015	ACLED (2015)
Water-related conflicts (selected examples)		
Territorial water disputes killed 5 and displaced many in Lake Chad	15/5–24/7, 1981	ICB (2015)
Fierce battle over the ownership of new islands as a result of falling water levels of the Lake (84 killed)	18/4–11/7, 1983	ICB (2015)
Clashes between two villages in Bol, Lake Chad over ownership of water points due to droughts/water scarcity (11 killed)	14–15/5, 1995	SCAD (2015)
Warring tribes clash over waterholes/wells/boreholes near Lake Chad areas of Djedaa and Massokory	20–21/11, 2000	SCAD (2015)
Farmers attacked herders after a herd of cattle wandered into cropland in search of water and pasture (8 death)	4–10/1, 2001	SCAD (2015)

Stresses are captured at the Lake Chad regional scale to highlight the exposure of locals to climate and insecurity. Respondents in our study areas were asked to give their perceptions about these stresses which were captured in our double exposure index

### The CWCVI framework approach

Climate–water conflict vulnerability was assessed based on the broader discourse on livelihoods (Ellis 2000), vulnerability (Füssel 2007) and the security consequences of climate change on human well-being (Adger 2010). We couch the composite index within the double stressor/exposure framework (Leichenko and Brien 2008) which emphasises the importance of dissecting the underlying contexts (using a contextual vulnerability interpretation (cf. O’Brien et al. 2007)) in which vulnerability is experienced, including how adaptation outcomes may reduce or amplify vulnerability (Silva et al. 2010).

We account for the security aspect of the double exposure framework by applying aspects of Busby et al.’s (2014) framing of climate security vulnerability, where vulnerability is conceived as a condition where people could be susceptible to death as a result of exposure to climate-related hazards. However, this past study lacks a bottom-up livelihoods approach. Instead, we frame climate–water conflict vulnerability as the propensity to be constrained by conflict-structured water threats as a result of climate stress. This encompasses situations where human populations are at risk of losing their livelihoods, including loss of life. Assessment of vulnerability in this context opens up considerations for a human security perspective in which attention is given to understanding biophysical exposures and socio-economic strategies to assist vulnerable populations from threats that limit their livelihoods and freedom (Adger 2010; Mason et al. 2011).

### Index computation

**Fig. 1:**
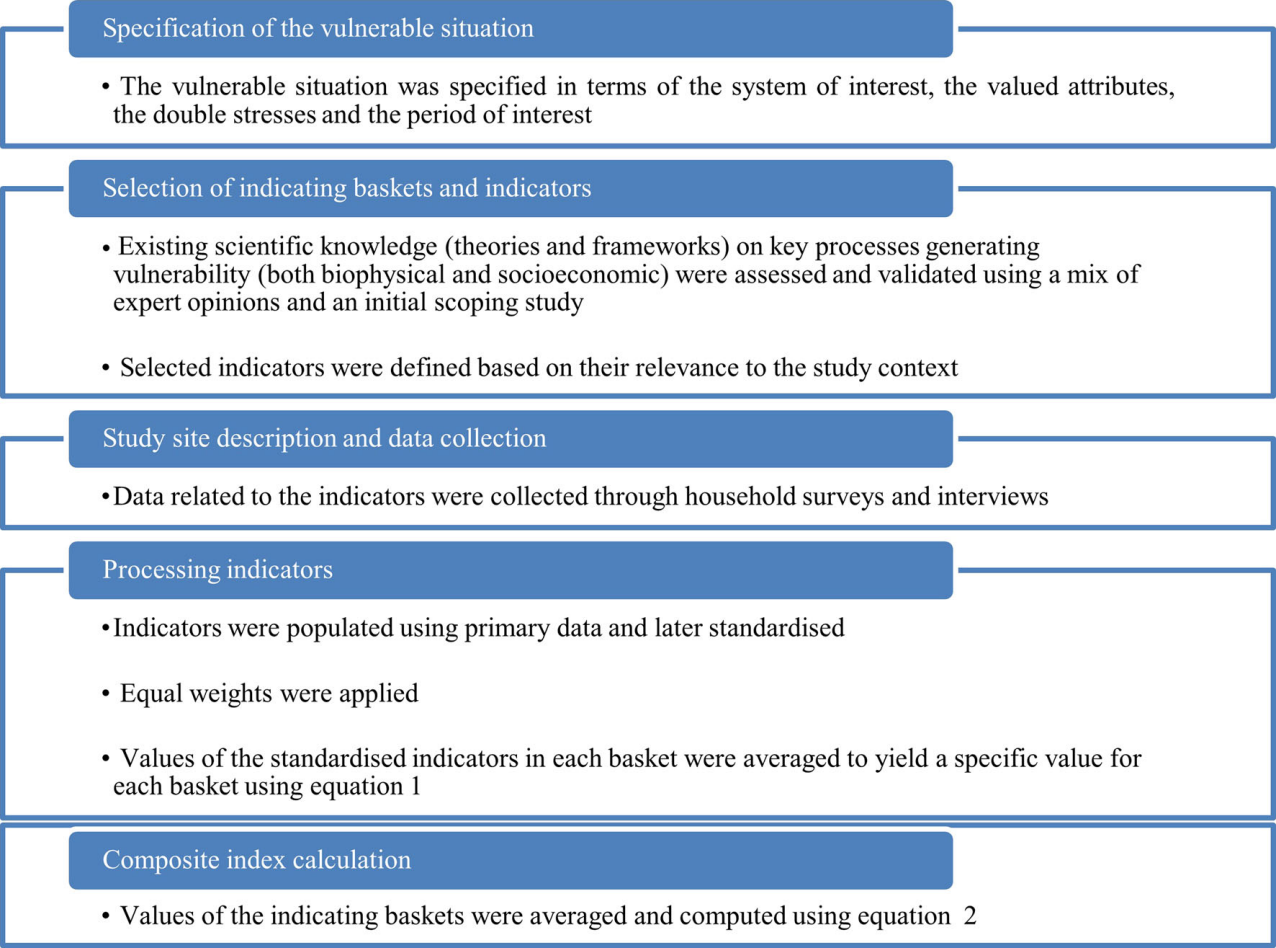
Methodological approach

**Fig. 2:**
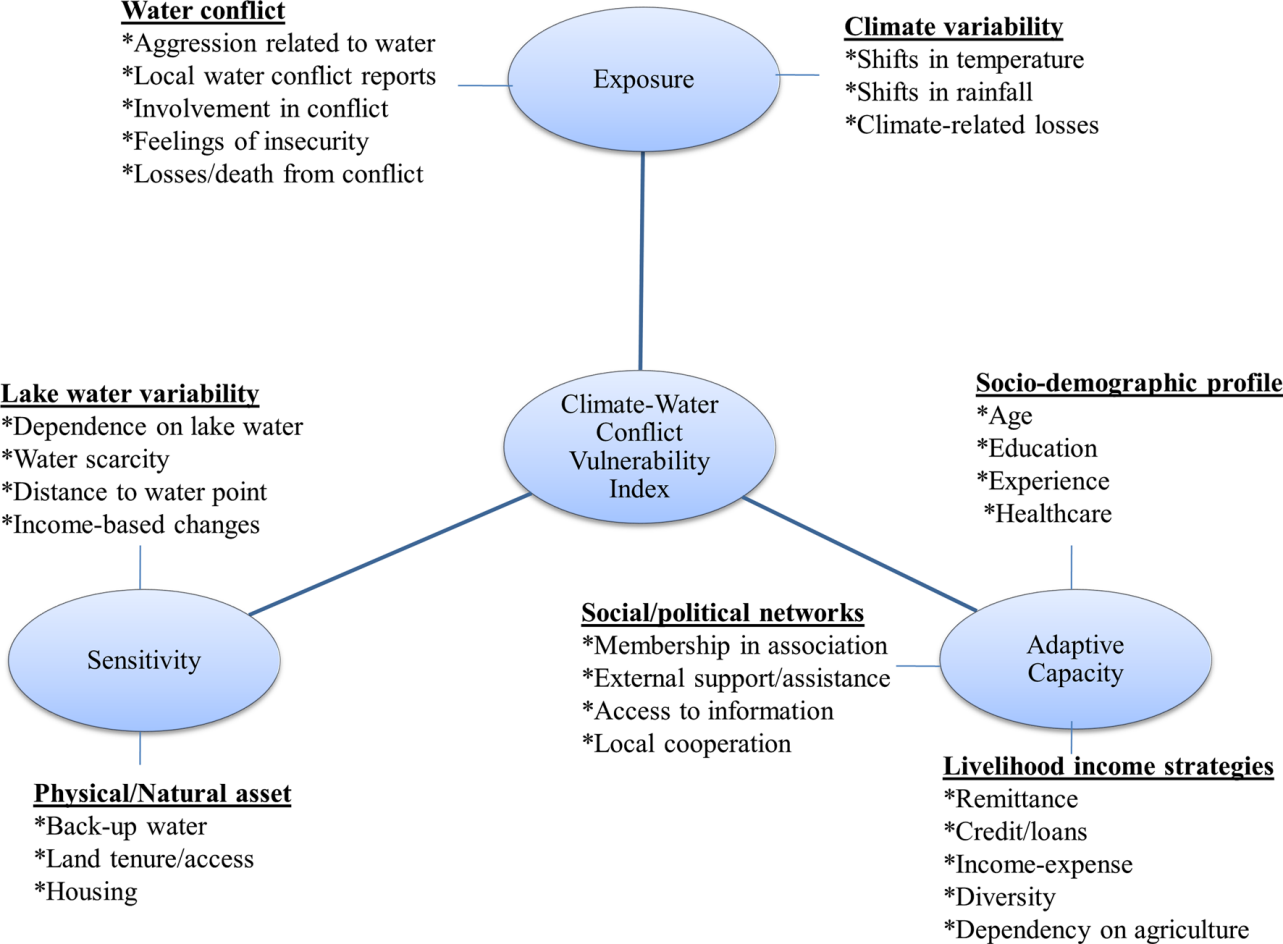
Indicative composite framework used to assess climate–water conflict vulnerability. *Indicators that are captured in each basket

We adopt a five-step interrelated process to compute the CWCVI (Fig. 1). Based on Füssel’s (2007) suggestion for describing a vulnerable situation, we identify the ‘human–environment system’ as our system of interest. By conceiving climate variability and water conflict as human well-being and livelihood security challenges, we identify the system’s valued attributes as ‘livelihoods and human well-being’ and the stresses of interest as ‘climate variability and water conflict’. For the ‘time period of interest’, we focus on a static snapshot of ‘current’ differential vulnerabilities occurring during 2009–2014, as vulnerability at the household level tends to be more dynamic than at national level (Eakin and Bojórquez-Tapia 2008). Resource user groups in the study villages constitute the unit of analysis. We utilise the IPCC’s tripartite typology of exposure, sensitivity and adaptive capacity (IPCC 2007) as a simple entry point for expressing vulnerability. We incorporate this typology in our categorisation scheme (Fig. 2) to identify seven indicating baskets that we consider relevant to operationalising vulnerability: exposure to (1) climate variability and (2) water conflict; sensitivity to (3) lake water variability and (4) physical/natural assets; and adaptive capacity captured by (5) socio-demographic profile, (6) livelihood income strategies and (7) social/political networks.

Indicators were selected deductively based on a review of the literature considering a broad spectrum of social and environmental challenges facing Lake Chad (e.g. UNEP 2004; Luxereau et al. 2012; Ovie and Emma 2012). Indicators were validated through consultations with Lake Chad Basin Commission staff and other professionals with specialist knowledge on the study themes. We incorporated the selected indicators into our questionnaires and confirmed the practicality of collecting the needed data through an initial field visit in July 2013. The supplementary material outlines how each indicator was quantified, the rationale for selecting each, as well as the survey questions used to collect the data associated with each indicator.

Raw household survey data were transformed into appropriate measurement units (percentages and indices) used to quantify the indicators. The CWCVI uses indicators measured on different scales. To bring the indicators to a uniform, comparable scale and allow for aggregation into a single index, standardisation was necessary (OECD 2008). We use a maximum–minimum (percentage ranked) transformation approach (Hahn et al. 2009) to capture the actual score of an indicator relative to the maximum and minimum spread of the entire range of values for that indicator. This was computed by obtaining the quotient of the difference between each actual indicator score and the minimum value of that indicator and the difference between the maximum and minimum values obtained from the total sample.

Weights are an important aspect of indexing approaches (see Barnett et al. 2008; Hinkel 2011; Wolf et al. 2013). Although what constitutes an appropriate weighting system can vary significantly based on contexts (Chen and Lopez-Carr 2015), we applied the balanced/equal weights framework used in Hahn et al. (2009) assuming that each indicating basket contributes equally to a group’s overall vulnerability despite that the number of indicators under each basket differs. Although this approach is adjustable, for example, to reflect the judgement of experts and values of groups in a participatory method (Eakin and Bojórquez-Tapia 2008), or by using the principal component analysis method (Abson et al. 2012), we regard our scheme as appropriate for conflict-prone settings where data are relatively difficult to gather and comparison is focused on groups perceived to be similarly exposed.

Finally, we calculated the value for the baskets by taking the average scores of the standardised indicators in each basket using Eq. 1:

1 $${\text{Indicating basket value}} = \left[ {\frac{{{\text{Indicator}}_{1} + {\text{Indicator}}_{2} + \cdots + {\text{Indicator}}_{\text{n}} }}{\text{n}}} \right]$$

where n represents the number of indicators for a particular basket.

The values of the indicating baskets were calculated to obtain the CWCVI score for each livelihood group (Eq. 2).

2 $${\text{CWCVI}}_{l} = \left[ {\frac{{{\text{w}}_{1} {\text{B}}_{1} + {\text{w}}_{2} {\text{B}}_{2} + \cdots + {\text{w}}_{\text{n}} {\text{B}}_{\text{n}} }}{{{\text{W}}_{1} + {\text{W}}_{2} + \cdots + {\text{W}}_{\text{n}} }}} \right]$$

where CWCVI is the computed index for livelihood group *l*, B_1_…..B_n_ are the indicating baskets, and w_1_……w_n_ represent the number of indicators in each basket. The value for each basket and overall vulnerability score were computed for each livelihood group (farmers, fishermen and pastoralists). The CWCVI is scaled from 0 (least vulnerable) to 1 (most vulnerable).

### Household surveys and interviews

Our study location was stratified into villages that are internally homogenous and externally heterogeneous based on a set of ‘screening’ criteria that emphasised lake dwellers’ major livelihood activities in terms of contribution to income and labour investments. We focused on three subgroups and adopted a United Nations (2008) sample size calculation method. At the 95 % level of confidence, a design effect of 2 to account for stratified sampling,^2^ ±10 % precision and a 50 % default value for point prevalence of selected indicators, we selected 240 respondents,^3^ composed of farming (*n* = 80), fishing (*n* = 80) and pastoral (*n* = 80) households, across seven villages of different sizes. Selected households represent approximately 43 % of households in each village.

Fieldwork was conducted using household surveys and semi-structured interviews. Due to the transient lifestyle of many households and the non-availability of a sampling frame for each village, we combined random walk, quota and snowball sampling techniques to select respondents (United Nations 2008). The survey process began at the homes of the village leaders, where consultations and permissions were obtained. To specify the paths of travel, some geographic starting points for each village were identified and randomly selected (i.e. farmlands, pasture areas, homesteads/settlements, waterways and desert-grazing sites) with assistance from a local guide/gatekeeper. Qualifying households from each subgroup participated in the survey until a predetermined quota (determined based on village sizes) was reached for each village and for each subgroup (see Table A5 in the Supplementary Material). Respondents were surveyed/interviewed at different locations—some in their farmlands, others while grazing animals or sorting fish from nets, and yet others in their homes. We applied snowball sampling to ensure low missing response frequencies and to attain a specific quota per village by requesting the village leaders and local guards to recommend households.

Data were collected following specific questions associated with each indicator and focused on current vulnerability concerns. Surveys and interviews were conducted in Arabic, French and Hausa and translated at the time of collection. Because of the socio-cultural and religious beliefs of the villages in which only males have the freedom to grant interviews, only responses from male household heads were recorded. Where the household head was unavailable, another male adult household member participated. Data analysis was conducted at the household level and later aggregated to obtain information on the different subgroups (farmers, fishers and pastoralists). Data were coded and analysed using SPSS v21.

### Limitations of the CWCVI approach

Our non-random sampling approach accounts for the transient lifestyle of many respondents. This limits our ability to comment on whether or not differences in vulnerabilities for farmers, fishermen and pastoralists are statistically significant (United Nations 2008). Nonetheless, the assignment of directionality from least to most vulnerable provides a straightforward alternative to compare and understand differential vulnerabilities (Hahn et al. 2009). While we recognise local arrangements that limit females from granting interviews, our data may appear to have under-represented vulnerable female-headed homes. In this case, we cannot comment on the magnitude of any potential selection bias. Further, because indicators are aggregated at the ‘livelihood group’ scale and averaged into one major indicating basket score, indexing does not emphasise differences within groups (e.g. between farmers or between fishermen). Also, the study does not ‘statistically’ account for the directionality of the relationship between indicators and vulnerability, although previous studies assume both exposure and sensitivity indicators to be positively correlated with vulnerability (Ide et al. 2014; Krishnamurthy et al. 2014). The weighting method applied constitutes less burden and time constraint on respondents and enabled us to avoid any complications that may result from experts’ inability to reach agreements over roles of indicators/baskets in vulnerability outcomes. Yet it is possible that other types of weights (or a combination of weighting schemes) could add confidence to the CWCVI. While many processes for operationalising vulnerability (particularly the schemes for selecting, validating, standardising and weighing indicators) involve normative judgement, the underlying approach employed to obtain different vulnerability scores here is consistent with the indexing approaches from larger vulnerability studies that utilise indicators (Brooks et al. 2005; Eakin and Bojórquez-Tapia 2008; Chen and Lopez-Carr 2015).

## Results

### CWCVI: farmers, fishermen and pastoralists

Values for each indicating baskets and the composite CWCVI for farmers, fishermen and pastoralists are presented in Table 2. This study sought the experience and views of farmers, fishermen and pastoralists in the surveyed villages to understand climate and water conflict exposures and vulnerability. Shifts in temperature and rainfall indices were generally similar for all livelihood groups. However, fishermen showed greatest vulnerability on the climate variability basket than farmers and pastoralists because of the reported higher climate-related losses due to the ‘low-fish-catch’ consequences from the direct impacts of climate parameters on the Lake Chad waters (CV_fishermen_ 0.993, CV_farmers_ 0.987, CV_pastoralists_ 0.963). The climate variability index serves to complement existing data on climate and therefore should be interpreted with caution since locations around the shores of Lake Chad are equally exposed to climate variability.

**Table 2:** Indexed indicating baskets and overall CWCVI scores for farmers, fishermen and pastoralists in the south-eastern portion of Lake Chad in Chad Republic

Indicating baskets	Number of indicators	Values for indicating baskets		
		Farmers	Fishermen	Pastoralists
Climate variability (CW)	3	0.987	0.993	0.963
Water conflict (WC)	5	0.768	0.352	0.750
Lake water variability (LWV)	4	0.495	0.495	0.573
Natural/physical assets (NPA)	3	0.387	0.863	0.847
Socio-demographic (SD)	4	0.450	0.475	0.470
Livelihood strategies (LS)	5	0.648	0.620	0.70
Social/political networks (SPN)	4	0.623	0.533	0.74
CWCVI		0.62	0.59	0.71

The aggression index was higher for pastoralists (0.98) than the other groups (farmers 0.81, fishermen 0.55). Pastoralists are often more aggressive during periods of extreme water and pasture shortages. Their involvement in water conflict often has a link with their inability to prevent straying animals from water points around farmlands or areas where fishermen’s nets or hook traps are positioned. Farmers (95 %) and pastoralists (96 %) reported more conflicts in their villages than fishermen (78 %). The feeling of insecurity index showed a greater vulnerability score for farmers (0.84) and a lower score for fishermen (0.09) compared to pastoralists (0.43). Farmers on average suffered greater losses in terms of crop destruction, post-harvest damages, money expended settling conflict cases in police stations, market closures and deaths due to water-related conflicts. This is reflected in the index for losses/death from conflict: farmers 0.65, pastoralists 0.52, fishermen 0.16. Overall, farmers were more vulnerable than pastoralists and fishermen on the water conflict basket (0.768 vs. 0.750 vs. 0.352, respectively).

The influence of the variability in Lake Chad waters on livelihoods has been systematically investigated elsewhere (see Okpara et al. 2015). However, pastoralists showed greater vulnerability on the lake water variability index (0.573) than farmers and fishermen who had identical scores of 0.495. A higher percentage of fishermen reported relying solely on Lake Chad waters for domestic and livelihood activities (lake water dependency index: fishermen 0.98, farmers 0.73, pastoralists 0.16). Consequently, many fishermen had experienced income-related changes resulting from the falling water levels of the Lake (index on income-based changes: fishermen 0.73, farmers 0.59, pastoralists 0.39). The high vulnerability score for pastoralists for this basket is reflected in the indicators that report water scarcity (0.94) and distance (over 50 km) to the Lake Chad water point (0.80).

The vulnerability scores for the physical/natural asset basket were similar for fishermen and pastoralists (0.863 versus 0.847). Both had a higher score than farmers (0.387). While most farmer respondents (90 %) have relatively consistent water supplies or a backup water source through village water pumps and private wells, fishermen and pastoralists reported a declining trend in the volume and quality of the water sources they can access (mostly rivers and streams around villages). Private land ownership is more common amongst farmers than the other groups with higher vulnerability scores for the land access indicator. Weak, less climate-resistant houses are common in all villages. Households live in either mud-walled thatched houses, brick houses with iron sheets or make-shift houses. The latter is common amongst pastoralists. Basic government-owned physical assets (schools, hospitals, boreholes, markets and telecommunication) are either non-existent or widely dispersed and poorly equipped.

Approximately 93 % of pastoralists, 86 % of farmers and 80 % of fishermen do not have access to social/political support during difficult times. Although fishermen are more isolated in terms of their village settings on islands, they often received more visits from NGOs, researchers and institutions. This contact enabled access to weather and livelihood-related information (without access to information index: pastoralists 0.68, farmers 0.63, fishermen 0.36). However, where promises regarding aid/support are made, they are often never fulfilled (personal communication with the leader of fishermen, Kaesai, February 2014). Except for a few farmers, the majority of respondents are not members of any formal local association. Cooperation was common amongst fishermen during periods of harsh weather conditions and aggression. Overall, pastoralists were more vulnerable than farmers and fishermen on the social/political network basket (SPN_pastoralists_ 0.74, SPN_farmers_ 0.623, SPN_fishermen_ 0.533).

Pastoralists showed greater vulnerability on the livelihood strategies basket (0.70) than farmers (0.648) and fishermen (0.620). Most farmers reported not receiving remittances in the form of cash and in-kind help from family members who travel outside the village to work or from friends/colleagues living mainly in urban areas (remittance index: farmers 0.78, pastoralists 0.63, fishermen 0.55). Further, the majority of fishermen reported that they have no access to credit/loans to support their activities, while more pastoralists reported having less income to cover important household expenses. A large proportion of farmers and pastoralists rely solely on one agriculture-based activity for income (agriculture dependency index: farmers 0.80, pastoralists 0.80, fishermen 0.61). Fishermen are more diversified in their livelihood activities; they fish, grow crops, trade fish, use boats for transportation and engage in menial jobs as ways to cope with livelihood challenges. The livelihood diversification scores reflect the vulnerability of the three groups (farmers 0.33, pastoralists 0.33, fishermen 0.28). When the five indicators were averaged, the vulnerability score for the livelihood strategies basket was highest for pastoralists.

The age index was highest for farmers (0.27) than fishermen (0.24) and pastoralists (0.18). Overall however, fishermen showed greater vulnerability on the socio-demographic basket than the other groups (SD_fishermen_ 0.475, SD_pastoralists_ 0.470, SD_farmers_ 0.450). A large proportion of household heads across all villages never attended school, although they reported having various years of experience in agricultural activities (farmers 16.8 ± 12.7; fishermen 14.2 ± 5.6; pastoralists 27 ± 8.1). Approximately 3 % of farmers reported having 0–2 years of experience. Over 90 % of fishermen and pastoralists have no access to medical services/facilities. During illness, they travel 2–12 kilometres to Guitte or Dandi to local clinics.

**Fig. 3:**
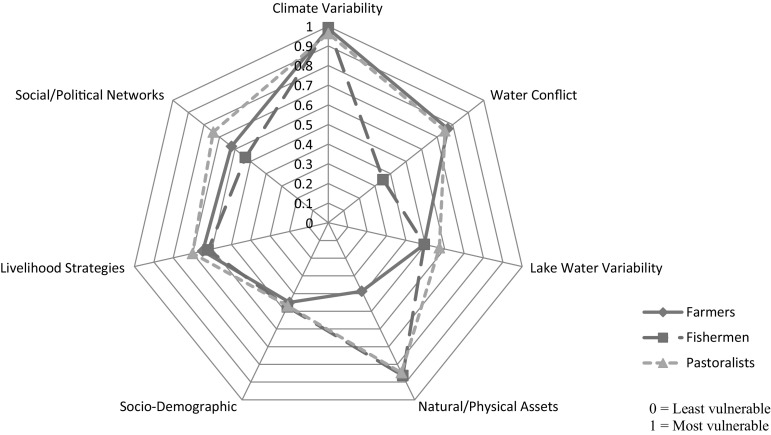
Vulnerability radar chart of the indicating baskets of the CWCVI for different livelihood groups at the south-eastern Lake Chad shores

Values for the indicating baskets are shown in a radar chart (Fig. 3). The diagram, with scales in 0.1 increments ranging from 0 (least vulnerable) at the centre of the web to 1 (most vulnerable) at the outside edge, shows which baskets contribute most to climate variability–water conflict vulnerability across the surveyed livelihood groups. Pastoralists are ‘most vulnerable’ in terms of Lake water variability, livelihood strategies and social/political networks, while farmers are ‘most vulnerable’ in terms of water conflict and fishermen in terms of climate variability, physical/natural assets and socio-demographic profile. In sum, pastoralists had the highest CWCVI (0.71) than farmers (0.62) and fishermen (0.59), indicating relatively greater vulnerability to climate variability and water conflict.

### The CWCVI and double exposure

**Fig. 4:**
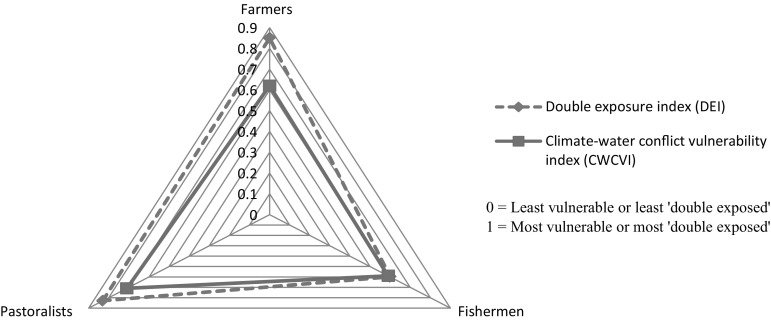
Integrated vulnerability and double exposure triangle diagram illustrating the CWCVI and DEI for farming, fishing and pastoral livelihood groups

Based on the IPCC vulnerability typology, eight indicators fall within our exposure categorisation (see Fig. 1). The CWCVI analysis captures double exposure in the form of climate variability and water conflict. The values of these two baskets, drawn from their contributing indicator scores, are incorporated into the double exposure index (DEI) computation to specifically draw out double exposure for all resource user groups. Table 3 shows the DEI for the different groups as DEI_farmers_ 0.85, DEI_pastoralists_ 0.83 and DEI_fishermen_ 0.60. Figure 4 illustrates an integrated vulnerability and ‘double exposure’ triangle which plots the scores for DEI and CWCVI for the three livelihood groups. Accounting for the DEI as an embedded component of the composite CWCVI, Table 3 indicates that farmers may be more exposed to the double (combined) effects of climate variability and water conflict than other livelihood groups in a context where the CWCVI was highest for pastoralists, and the CWCVI and DEI for fishermen yielded similar values.

**Table 3:** Summary of computed double exposure indices for farmers, fishermen and pastoralists

Based on the summarising method^a^: ${\text{V}}_{\text{DE}} = {\text{DEI}} = \,\left[ {\frac{{({\text{W*B}})_{\text{CV}} + ({\text{W*B}})_{\text{WC}} }}{{{\text{W}}_{\text{CV}} + {\text{W}}_{\text{WC}} }}} \right]$	DEI_farmers_	$\frac{3(0.987) + 5(0.768)}{3 + 5} = 0.85$
	DEI_fishermen_	$\frac{3(0.993) + 5(0.352)}{3 + 5} = 0.60$
	DEI_pastoralists_	$\frac{3(0.963) + 5(0.750)}{3 + 5} = 0.83$

V_DE_ is a recast version of Eq. 2 (adopted from Hahn et al. (2009)) accounting for vulnerability under double exposure. DEI is double exposure index. W (number of indicators in each basket) and B (indicating basket) are based on climate variability (CV) and water conflict (WC) contributing indicators

^a^Index values are interpreted as relative values for livelihood groups within the study context only and are based on views from the local resource users in our sample. The DEI is on a scale from 0 (least ‘double exposed’) to 1 (most ‘double exposed’)

## Discussion

### Unpacking the implications of the CWCVI–DEI assessment

Recurrent shifts in temperatures and rainfall, including water-related conflicts, are well-known livelihood stresses in the Sahel, often acting in combination to alter agricultural production, food supplies and livelihood dynamics (Benjaminsen 2008; Couttenier and Soubeyran 2014; Uexkull 2014). Losses from climate and water conflict impacts represent an important vulnerability concern in our study context. In contrast to existing data on temperature and rainfall which suggest similar climatic patterns for locations within the Lake Chad basin, local people’s perceptions about climate variability indicated that differences exist in local exposures, vulnerability and responses. Our findings show that fishermen are more vulnerable to climate-related losses, but were better off in terms of response capacities through social/political networks and livelihood income strategies than farmers and pastoralists. Most local water conflict reports were received from pastoralists, including reports on aggression related to water. Yet it was the farmers who suffered the most from the consequences of water conflict. The high percentage of farmers who felt insecure in their villages and who reported crop, cash and human losses in the past five years underline the reasons many entry routes into farm villages were manned by local security personnel. The majority of farmers reported having the mobile telecommunication contacts of security officials to enable receipt of immediate help in the event of conflict.

Although our analysis yielded a high water conflict vulnerability score for farmers, we noted low vulnerability on the natural/physical asset basket for farmers, particularly in terms of access to backup water sources and land. This reflects why pastoralists (mostly) often encroached into farmlands and/or migrated towards farm villages. Resource scarcity and the relatively regular contacts farmers have with pastoralists and fishermen underlie the reasons for approximately 75 % of the conflicts reported in farm villages. This finding has crucial implications for conversations regarding Lake Chad variability. Although the Lake waters play a central role in livelihoods, the relatively high dependence of villages on the Lake contributed to the income-based changes they experienced during low water levels. While fishermen suffered from limited water quality, pastoralists reported that they struggle to find water (in terms of volume and quality) during annual dry periods. Although pastoralists did not report the same level of dependence on lake waters and income-related changes resulting from lake water fluctuations as other groups, they had a higher vulnerability to lake water variability. Development programmes for village assistance regarding water supplies might constitute an appropriate intervention for locals, especially pastoralists needing secure watering points. When such an intervention is locally defined and centrally enforced, the frequent aggression amongst resource users during periods of water shortages can become minimal (Turner 2004).

Despite receiving more remittances than farmers and having greater access to credit/loans than fishermen, the pastoralists showed more vulnerability than the other groups on the livelihoods income strategies index. Depending solely on livestock for meat, milk and cash meant that pastoralists are prone to income fluctuations resulting from cattle devaluation, diseases, scarcity of quality feed and conflict (Majekodunmi et al. 2014). Opportunities for alternative and supplementary livelihoods were limited in all the surveyed villages. The low socio-demographic profiles, as reflected mostly through limited education amongst a large proportion of the respondents, suggest why efforts by a few to diversify agricultural livelihoods were unable to fill immediate cash needs. To better capture livelihood income, future research might approach this by including quantitative estimates of annual income and expenditure across various groups.

While there are many measures of social/political networks at the local level (Eakin and Bojórquez-Tapia 2008; Hahn et al. 2009), documenting membership in associations, receipt of external support/assistance, access to climate and livelihood-related information and local cooperation provide an indirect way of teasing out the contribution of social/political networks to differential vulnerability across different livelihood groups (Shah et al. 2013). Membership in group- or village-level associations influences the way local people bond with one another, including their access to informal insurance and logistic supports, and capacity for collective actions (Baird and Gray 2014). This form of social capital is crucial for decreasing vulnerability to climate and conflict impacts (Uexkull 2014). In contrast to fishermen who were better off in terms of cooperation and access to information, and a few farmers who belonged to farming associations, pastoralists were more limited in their social/political networks. Pastoralists’ migratory lifestyle influences their perception of the cost of and benefits from social/political participation or engagement with authorities at the village and district levels (Thébaud and Batterbury 2001). Despite occasional visits by agencies providing social and economic assistance, respondents reported that such visits were yet to translate into any solid relationship between villages and agencies/institutions. Further investigation (e.g. through focus groups) into how location-specific characteristics disrupt village linkages with the state and aid donors would help uncover reasons why social support and livelihood assistance remain largely non-existent in the surveyed villages.

Our interactions with local experts and observations during field visits suggest that water conflict may have contributed more to local exposure challenges than climate variability. While this may not be detected from the DEI scores, the DEI nonetheless reflected an important conclusion regarding climate variability (CV) and water conflict (WC) exposures amongst different groups in the area. Despite the high climate-related losses reported by fishermen, including their high vulnerability to natural/physical assets and socio-demographic profile, they showed lower CWCVI and DEI scores than the rest of the groups. Indicators that constitute our sensitivity and adaptive capacity elements did not create any difference between ‘double exposure’ and vulnerability in our computation of fishermen’s vulnerability. This might require further investigation to understand why this is the case. Fishermen’s low vulnerability to water conflict and better social networks may have accounted for the low CWCVI score. Although farmers and pastoralists did not show similar low vulnerability, in the absence of development supports that address poor infrastructure, lack of representation and ineffective systems of conflict management, including social protection and livelihoods planning (Luxereau et al. 2012; Ovie and Emma 2012), the local populations in Lake Chad would face challenges in adapting to future changes in livelihood conditions.

### Prioritising vulnerability assessment in climate and water conflict research

There is a livelihood security imperative to frame climate conflict research around vulnerability (Gemenne et al. 2014). Yet applying a vulnerability lens to explain climate and water conflict link raises complex challenges. The link is not exclusively a collection of environmental (supply), institutional (restraint) and social (demand) drivers that can be understood purely in scientific or technical one-size-fits-all ways (Böhmelt et al. 2014). It reflects a conundrum of underlying realities that are context, place and time specific, and contingent on an array of theoretical postulations regarding what indicators or metrics that researchers deem important (Buhaug 2015). This is why attempts to link climate and conflict stresses in vulnerability assessments is arguably the least advanced aspect of vulnerability science (Busby et al. 2014). Nevertheless, the contextual nature of the CWCVI provides a vulnerability lens depicting a range of indicating variables that inform climate–water conflict thinking for lake-dependent environments. It points to a repertoire of potential explanatory factors (e.g. feelings of insecurity, dependency on Lake water and agriculture, climate-related losses and livelihood diversity) linking climate and (water) conflict (Scheffran et al. 2012).

Our findings underline how background conditions of vulnerability are an important entry point in identifying ways people are likely to face threats of death or livelihood emergencies resulting from climate-related events. Theoretical conversations on peace building show that conditions where human needs are grossly denied can be critical drivers of vulnerability (Le Billon 2003; Yardley 2013; Matthew 2014). Territories with problematic societal conditions such as insurgencies, high levels of militarisation and increased displacement of human population convey a broad spectrum of leading conditions that shape the climate conflict dimensions of vulnerability (Verhoeven 2014). Although the arrival of water conflict is often signalled years in advance by deteriorating climatic, socio-demographic, economics and governance conditions, the CWCVI variables provide a basis for a fine-grained causality analysis that can lead to socially focused solutions—such as group agricultural cooperatives, conservation of common property resources and conflict resolution, and strengthening of collective adaptation actions. These solutions are consistent with what many consider as suitable vulnerability interventions in a climate–conflict context where vulnerability is experienced (Scheffran et al. 2012; Sterzel et al. 2014).

Our results establish that biophysical and socio-economic factors trump several determinants of vulnerability. Comparison with climate and water conflict case studies (e.g. Ludwig et al. 2011; Tir and Stinnett 2012; Böhmelt et al. 2014; Kuzdas and Wiek 2014; Selby and Hoffmann 2014; Ide 2015) indicates both good agreement in terms of the utility of our indicators in understanding climate variability and water conflict links and a prospect to expand the indicators as data for other conflict-torn portions of the Lake become available.

It is important to stress that research efforts to prioritise vulnerability frameworks or indicators applicable to climate and water conflict analysis should be undertaken with caution. Choice of vulnerability indicators is largely based on subjectivity and use of several kinds of proxies (Hinkel 2011) that may influence how climate–water conflict relations are interpreted. By using a mix of expert views and theories, our study has demonstrated the need to control the way normative judgements translate into indicating variables used in characterising vulnerability to double stresses. Further, the directions of causality, in terms of pathways and feedbacks, may not be easily teased out from quantitative, empirical vulnerability studies. Additional steps in econometric modelling (e.g. Opiyo et al. 2014) underpinned by fundamental variables that are known to influence the directionality of vulnerability may complement indicator-based approaches. In doing this, the research design can move beyond mainstream views that privilege climate-induced resource scarcities in conflict outcomes by considering the balance between vulnerability and adaptability as a key contextual entry point to understanding climate–water conflict relations.

## Conclusion

Many works on climate and water conflict relations pay insufficient attention to vulnerability determinants, in particular the fundamental issues that shape directionality of vulnerability. Although the contested nature of the vulnerability concept is widely recognised, it cannot be assumed that there is broad consensus regarding what constitutes scientifically sound explanatory variables for climate and water conflict relations. In response to recent calls to uncover local dynamics of climate and environmental conflict interactions (Gemenne et al. 2014; Böhmelt et al. 2014), we present the CWCVI as a tool for exploring ordinary people’s differentiated vulnerability and capacities to adapt to change. The tool resonates with livelihood perspectives and uses a normative framing consistent with the context, place and time-specific nature of both vulnerability and climate conflict analyses.

We applied the CWCVI in selected Lake Chad villages composed of farming, fishing and pastoral livelihoods and found that in contrast to farmers and fishermen, pastoralists were more vulnerable to climate variability and water conflict stresses. They were prone to climate-structured aggressive behaviour, have limited social networks and livelihood income strategies, and their migratory lifestyle often pitched them against other resource users. Using ‘views from the vulnerable’ and accounting for the DEI as an embedded component of the CWCVI, we illustrated that water conflict and climate variability are important exposure elements amongst groups and that farmers may be more exposed to the double (combined) effects of climate variability and water conflict. Further, we employed the CWCVI to understand how drivers of vulnerability may be useful in explaining climate and water conflict interactions and noted that besides informing climate conflict thinking, the CWCVI can provide the basis for causality analysis. It privileges the directionality of vulnerability by focusing on the usefulness of the vulnerability lens over resource scarcity in operationalising climate–water conflict relations for lake-dependent environment.

The CWCVI and DEI approaches have several strengths. First, our multi-step methods of index computation and data collection provide detailed quantitative information about livelihood vulnerabilities, as well as local perceptions of shifting climatic conditions and conflict outcomes. Many indicator-based vulnerability studies focus on quantitative comparison of vulnerabilities across districts and regions, emphasising a single environmental or social stressor/hazard. Few studies use household survey data to develop vulnerability indices that capture double exposures across different resource user groups in the manner this study has done. Second, the DEI approach uses the views of ‘vulnerable locals’ to gain insight into climatic and conflict situations and therefore can comment on differences in double exposure amongst farmers, fishermen and pastoralists despite popular belief (based on existing secondary data) that villages within Lake Chad are similarly exposed to climate and water conflict. Third, the study presents a model that aggregates indicators to better understand the strength of livelihoods/households to resist pressures resulting from double exposure. Although it is unclear how the CWCVI and DEI scores might change if different weighting methods are employed, comparison with other studies across the region confirms that fishing and fish trading allow for more stability (see Luxereau et al. 2012) and that the ‘capacity of fishing activities to generate instantaneous gains represent an enormous advantage over farming’ (Bene et al. 2003, p. 43) and over pastoral activities as well. This, somehow, confirms that the group we find to be relatively less vulnerable (i.e. fishermen) is the same one that comes up in these studies.

The deep-rooted issues identified through the CWCVI raise concerns about the ability of resource users to confront current and future challenges associated with climate change and growing insecurity. The CWCVI communicates locally appropriate insights about what may contribute to apparently new forms of interventions for rural livelihoods. Replication of our approach in the same location over time might communicate useful information about changes in vulnerability as adaptation and other livelihood interventions are initiated provided that any potential biases in sampling techniques and indicator selections are given considerations. However, as with any index approach, there is need for caution in interpreting any empirical findings since indicators and indices, by their nature, can mask underlying multidimensional realities shaping vulnerability.

Further refinement of the indicator framework might focus on regional contexts to more accurately quantify how the factors operating beyond the household realm shape the roles of climate and water conflict in driving local vulnerabilities. Similarly, future research can account for duration and severity of double exposure elements to uncover the extent indicators and indices oversimplify complex climate and water conflict realities. In doing this, scenarios of climate and conflict changes can be introduced into the indexing process to capture hidden and also future vulnerabilities. It is hoped that the CWCVI tool will help guide discussions on the need to prioritise vulnerability assessments in climate conflict research, particularly in order to better explain the interactions between climate variability and water conflict in a way that is easy to understand without glossing over the complexity.

## Electronic supplementary material

Below is the link to the electronic supplementary material.

Supplementary material 1 (DOCX 43 kb)

## Footnotes

1 The villages are Miterine and Guitte (farming villages), Kaesai, Basara and Kouri (fishing villages) and Dandi, Ngurutu and Guitte (pastoral villages). Guitte is a mixed farming and pastoral village. See Table A5 in the Supplementary Material.

2 We assume a sample design effect of 2, implying that the sample variance for the stratified sample is two times bigger than it would be if the surveys were based on a simple random sample of the same size.

3 Sample size formula: *N* = [(z^2^) (r) (1−r) (f) (k)]**/[**(p) (n) (e^2^)], *N* = sample size; *z* = 1.96 (95 % CI); *r* = 0.5 (default value for prevalence of indicators); *f* = 2 (sample design effect); *k* = 1; *p* = 0.54; *n* = 6; *e* = 0.05. Value descriptions/calculations are based on United Nations (2008 p. 41–43).
