# Supplementary material for: Using a novel climate–water conflict vulnerability index to capture double exposures in Lake Chad
Source: Reg Environ Change. 2016 Jul 6;17(2):351–66. doi: 10.1007/s10113-016-1003-6 (PMC7114970; doi:10.1007/s10113-016-1003-6)
Supplement: Supplementary file 1 — Supplementary material 1 (DOCX 43 kb) [file 10113_2016_1003_MOESM1_ESM.docx]

| Table A1.  The IPCC elements, indicating baskets and indicators | Data source^1^ |
| --- | --- |
| **Exposure**  1. Climate variability:  Shifts in temperature (% of households reporting long-term (≤ 20 years) shifts in temperature)  Shifts in rainfall (% of households reporting long-term shifts in rainfall)  Climate-related losses (% of households reporting losses –crop, livestock, fishery – due to droughts/floods) | HQs |
| 2. Water conflict:  Aggression related to water (% of households reporting aggression over water)  Local water conflict (% of households reporting water conflict)  Involvement in conflict (% of households reporting involvement in conflict over water)  Feelings of insecurity (% of households feeling insecure in village)  Losses/death from conflict (% of household reporting injury/losses/death due to water conflict) | HQs |
| **Sensitivity**  3. Lake water variability  Dependency on Lake water (% of households reporting Lake water as sole/nearest water source)  Water scarcity (% of households reporting water scarcity)  Distance to water point (% of households reporting long distance to water points ≥ 30 mins. walk)  Income-based changes (% of households reporting that the shrinking Lake has reduced their income) | HQs |
| 4. Physical/natural assets  Back-up water (% of households without backup for drinking/well water)  Land tenure/access (% of households that can’t access/own land – where to live, farm, graze)  Housing (% of households that own/live in weak, less climate resistant house) | HQs |
| **Adaptive capacity**  5. Socio-demographic profile  Age (% of households where head ≥ 50 years)  Education (% of households where head is not educated)  Experience (% of households where head has ≤ 2 years of experience in farming/fishing/herding)  Healthcare (% of household without access to healthcare) | HQs |
| 6. Livelihood income strategies  Remittance (% of households receiving no remittance since the past 24 months)  Credit/loans (% of households with no access to credit/loans)  Income-expense (% of households with less income to cover important expenses –food, clothing, housing)  Dependency on agriculture (% of households depending solely on one agro-based activity)  Diversity (average livelihood diversification index) | HQs |
| 7. Social networks  Membership in association (% of households that are not members of any group/association)  External support (% of household where village can’t access external supports at difficult times)  Access to information (% household reporting no access to climate/security/aid-support information)  Local cooperation (% of households reporting less cooperation among groups/village folks during scarcity) | HQs |

^1^ HQs = household questionnaires

Table A2.

Indicators, survey questions and rationale

| Indicating baskets and their associated indicators | Survey/interview questions associated with each indicator | Explanatory notes/rationale |
| --- | --- | --- |
| **Climate variability (CV)**  Shifts in temperature  Shifts in rainfall  Climate-related losses | Have you noticed any long-term shifts or changes (≤ 20 years) in temperature in your area?  Have you noticed any long-term shifts in rainfall in your area?  What changes has climate variability had on your livelihoods in terms of losses incurred in the past 5 years? | The CV basket captures local people’s knowledge/experiences of temperature and rainfall shifts. Local perceptions of climate variability often provide insights for adaptation planning (Tambo and Abdoulaye 2013). Water supply is highly dependent on air temperatures and rainfall (Bates *et al*., 2008). High temperatures and heavy rainfall influence crop productivity, livestock wellbeing (mobility, fertility) and fish harvest/diversity/distribution/abundance (Kotir 2011; Sarr 2012). Agriculture-related losses are a major driver of low income (Codjoe and Owusu 2011), and losses provide a proxy for exposure in our study context. The higher the proportional losses incurred, the higher the vulnerability to climate and water conflict. Greater losses imply limited capacity to manage double exposure. Losses incurred are qualitatively described in this study, and they include crop failure, dying animals, depleting fisheries and disruptions of settlement patterns. Percentages of households reporting climate variability are recorded and used in computing the CV index. |
| **Water conflict (WC)**  Aggression related to water  Local water conflict report  Involvement in conflict  Feeling of insecurity  Losses/death from conflict | Are farmers/fishermen/pastoralists aggressive about water conditions?  Are you aware of any water conflict that has turned violent in your area?  In the past year, have you been involved in any conflict related to water?  Do you feel secure in your village? (i.e. safe from threats of conflict and violence)  Have you suffered any injury/loss of family members, relatives or friends due to water conflict? | The WC basket captures the percentages of different households reporting aggression in terms of the urge to grab and control public water sources, confrontations and clashes at public water points; reports on local water conflicts that have turned violence, people’s involvement in conflict, feelings of insecurity in villages and losses/injuries/deaths related to water conflict. Water conflict is a stress factor within the vulnerability context (Hahn *et al*. 2009). Since local people cannot fight nature (i.e. changing patterns of water flows or supplies), they often turn it against one another (Funder *et al*. 2012). This is especially the case in locations where the importance of water in sustaining agricultural livelihoods provides a pathway to conflict (Conca 2006), and where rivers cross national boundaries and water institutions lack technical and administrative capacities to govern the use of water (Ludwig *et al*. 2011). Given the increasingly harsh climatic conditions driving water supplies, including the lack of a suitable substitute for water, water conflict remains a major exposure element in dryland Sahel region of Africa (IPCC 2014). |
| **Lake water variability (LWV)**  Dependency on Lake water  Water scarcity (volume)  Distance to water point  Income-based changes | Is Lake Chad the only water point/source to you? How long have you been using water from the Lake?  What problems do you experience generally in accessing water? Is scarcity of water a concern to you in this village as the Lake shrinks?  How far is your nearest water point? (distance ≥ 30 minutes walk is considered a long distance in our study)  Have there been any major changes in the benefits you derive from the Lake in recent years (i.e. since the past 5 years)? Indicate the extent to which the shrinking Lake is responsible for ‘reduced income’ in your household (Here, a scale of 1 – 5, from ‘to no extent = 1’ to ‘a very great extent = 5’, was used to quantify responses on income-based changes). | The LWV basket captures sensitivity in terms of Lake Chad water depletion. Lakes directly or indirectly support local livelihoods and their fluctuations provide a pathway to understanding the vulnerability of different resource user groups who rely on water sources flowing into and through lakes (Rast 2014). The Lake Chad basin connects many local migrants, and therefore represents an important factor in explaining climate influences and local water conflict events in the region (given the more than a decade variability (i.e. shrinkage) in the Lake’s water levels) (Onuoha 2009). |
| **Physical/natural asset (PNA)**  Back-up water  Land tenure/access  Housing | Do you have back-up for drinking water (e.g. access to private well/piped water)?  Does your household own/can access land?  What type of house do you have or live in? | As water becomes scarcer, households without back-up water facilities, both for domestic and livelihood activities, may likely become more sensitivity to water scarcity and struggles that characterise drought-ridden locations (Freitas 2013).  Households that do not own the land where they currently live/farm/graze animals (or are unable to access/rent land legally) are likely to be sensitive to changing climatic and conflict conditions (Butler and Gates 2012).  Local people who own or live in houses that are unable to withstand a severe climatic event (e.g. heavy rains or winds) will have their settlement patterns disrupted under climate change and demographic pressures (Paavola 2008; Geest and Warner 2015; De Souza 2015). |
| **Socio-demographic profile (SD)**  Age  Education  Experience  Healthcare | What is your age? (relatively less active groups are assumed to be ≥ 50 or ≤ 20 years of age)  What is your highest level of education?  How many years have you been into farming/fishing/animal herding?  Do you have access to healthcare in your village or elsewhere near your village? | The SD basket illustrates the adaptive capacity of the locals in terms of active age group, level of education, experience and health of household heads (Shah *et al*. 2013). In this study, we assume the active age groups to be individuals between 20 and 50 years old. Active in this sense implies capacity to do useful work (i.e. income-generating) and to pursue livelihood strategies in a harsh environment such as Lake Chad with limited income opportunities. The livelihood literature establishes that households headed by individuals in the active age bracket, with formal education (up to or above the secondary school level), having over 2 years of experience in agricultural activities and in good health, are likely to be relatively less vulnerable to external stresses, such as climate change (Scoones 2009; Sallu *et al*. 2010; Mubaya *et al*. 2012). |
| **Livelihood income strategies (LS)**  Remittance  Credit/loans  Income-expense  Dependency on agriculture  Diversity (livelihood diversification) | Have you received remittances in the last 24 months?  Do you have access to credit/loans for your household activities?  How would you rate your household income considering your expenses?  Do you work on other activities beside crop cultivation/fishing/  livestock herding?  Same as above | Remittances include money (cash) and in-kind goods received from family members, friends and colleagues living mainly outside the villages (Abdelali-Martini and Hamza 2014).  The indicator captures the percentage of households where the head is able to access credit facilities.  The scale used here are: 1 = usually not enough to cover important household expenses; 2 = just enough to cover important household expenses; 3 = usually have some left over after important household expenses have been met. Important household expenses considered here include food, medicine, clothes, education and shelter/housing.  The ‘other activities’ implied here include trading, civil or public services, bricklaying, transport (using motor bikes, boats or camels/horses) or self employment (private kiosks). Activities of these kind, including access to remittances and credit/loans, often account for household income (Hahn *et al*. 2009).  Number of activities determine how diverse a household head can be; and the more diverse households are, the more they are able to cope with perturbations and stresses (Paavola 2008). |
| **Social/political networks (SPN)**  Membership in association  External support/assistance  Access to information  Local cooperation | Do you belong to any social group/association?  Is your household/village able to access external support during difficult times?  Do you have access to information (climate, security and livelihood related information)?  Do groups/village folks cooperate more during water scarcity/water conflict? | The SPN basket captures the social contacts and assistance local people have which form part of their adaptive capacity (Goulden *et al*. 2013). The range of social capital organised within households, groups or across communities often constitute an effective tool for assessing local capacities to deal with sudden environmental and social changes (Nunan 2010; Baird and Gray 2014). |

Note: Perceptions about our indicators may differ from indicators collected through other processes in terms of local applicability, ease of collection (i.e. practicality of gathering primary data), ability to identify divergent opinions/values amongst local people and relevance to decision makers. The indicators we used to compute the CWCVI are based on conditional factors prevalent in our study location and may not apply to other settings/populations.

Table A3.

The computed values for each indicating baskets and indicators for south-east Lake Chad

| Baskets | Indicators | Farmers  Value _a_ Value_s_ | | Fishermen  Value _a_ Value_s_ | | Pastoralists  Value _a_ Value_s_ | |
| --- | --- | --- | --- | --- | --- | --- | --- |
| Climate variability |  |  | **0.987** |  | **0.993** |  | **0.963** |
|  | Shifts in temperature | 100 | 1.00 | 100 | 1.00 | 95 | 0.95 |
|  | Shifts in rainfall | 100 | 1.00 | 100 | 1.00 | 100 | 1.00 |
|  | Climate-related losses | 96 | 0.96 | 98 | 0.98 | 94 | 0.94 |
| Water conflict |  |  | **0.768** |  | **0.352** |  | **0.750** |
|  | Aggression related to water | 81 | 0.81 | 55 | 0.55 | 98 | 0.98 |
|  | Local water conflict reports | 95 | 0.95 | 78 | 0.78 | 96 | 0.96 |
|  | Involvement in conflict | 59 | 0.59 | 18 | 0.18 | 86 | 0.86 |
|  | Feeling of insecurity | 84 | 0.84 | 9 | 0.09 | 43 | 0.43 |
|  | Losses/death from conflict | 65 | 0.65 | 16 | 0.16 | 52 | 0.52 |
| Lake water variability |  |  | **0.495** |  | **0.495** |  | **0.573** |
|  | Dependence on lake water | 73 | 0.73 | 98 | 0.98 | 16 | 0.16 |
|  | Water scarcity | 57 | 0.57 | 27 | 0.27 | 94 | 0.94 |
|  | Distance to water point | 9 | 0.09 | 0 | 0.00 | 80 | 0.80 |
|  | Income-based changes | 59 | 0.59 | 73 | 0.73 | 39 | 0.39 |
| Physical/  Natural assets |  |  | **0.387** |  | **0.863** |  | **0.847** |
|  | Back-up water | 10 | 0.10 | 90 | 0.90 | 71 | 0.71 |
|  | Land tenure/access | 6 | 0.06 | 69 | 0.69 | 83 | 0.83 |
|  | Housing | 100 | 1.00 | 100 | 1.00 | 100 | 1.00 |
| Socio-demographic profile |  |  | **0.450** |  | **0.475** |  | **0.470** |
|  | Age | 27 | 0.27 | 24 | 0.24 | 18 | 0.18 |
|  | Education | 84 | 0.84 | 75 | 0.75 | 74 | 0.74 |
|  | Experience (≤ 2 years) | 3 | 0.03 | 0 | 0.00 | 0 | 0.00 |
|  | Healthcare | 66 | 0.66 | 91 | 0.91 | 96 | 0.96 |
| Livelihood income strategies |  |  | **0.648** |  | **0.620** |  | **0.70** |
|  | Remittance | 78 | 0.78 | 55 | 0.55 | 63 | 0.63 |
|  | Credit/loans | 70 | 0.70 | 90 | 0.90 | 81 | 0.81 |
|  | Income-expense | 63 | 0.63 | 76 | 0.76 | 93 | 0.93 |
|  | Dependence on agriculture | 80 | 0.80 | 61 | 0.61 | 80 | 0.80 |
|  | Diversity* | 0.464 | 0.33 | 0.424 | 0.28 | 0.466 | 0.33 |
| Social/  political networks |  |  | **0.623** |  | **0.533** |  | **0.740** |
|  | Membership in association | 55 | 0.55 | 84 | 0.84 | 64 | 0.64 |
|  | External support/assistance | 86 | 0.86 | 80 | 0.80 | 93 | 0.93 |
|  | Access to information | 63 | 0.63 | 36 | 0.36 | 68 | 0.68 |
|  | Local cooperation | 45 | 0.45 | 13 | 0.13 | 71 | 0.71 |
| Vulnerability | Climate-Water Conflict Vulnerability Index (CWCVI) |  | **0.62** |  | **0.59** |  | **0.71** |

Note: *All units are in percentages with maximum and minimum indicator values (across groups) given as 100 and 0 respectively, except for the ‘livelihood diversity index’ where the maximum and minimum values are 1.0 and 0.2 respectively – calculated following Hahn *et al*. (2009) as the inverse of the number of agricultural livelihood activities + 1, reported by each household.

V _a_ and V _s_ represent actual and standardised values respectively.

Table A4.

Indexed indicating baskets and overall CWCVI for farmers, fishermen and pastoralists in the south-eastern portion of Lake Chad in Chad Republic

| Indicating baskets | Number of indicators | Values for indicating baskets  Farmers Fishermen Pastoralists | | |
| --- | --- | --- | --- | --- |
| Climate variability | 3 | 0.987 | 0.993 | 0.963 |
| Water conflict | 5 | 0.768 | 0.352 | 0.750 |
| Lake water variability | 4 | 0.495 | 0.495 | 0.573 |
| Natural/physical assets | 3 | 0.387 | 0.863 | 0.847 |
| Socio-demographic | 4 | 0.450 | 0.475 | 0.470 |
| Livelihood strategies | 5 | 0.648 | 0.620 | 0.70 |
| Social/political networks | 4 | 0.623 | 0.533 | 0.74 |
| **CWCVI** |  | **0.62** | **0.59** | **0.71** |

Table A5.

Characteristics of study villages and household surveys undertaken

| Village surveyed | Location^b^ | Estimated* households | Estimated population size |  | Number of household heads sampled |
| --- | --- | --- | --- | --- | --- |
| Farming villages:  Miterine  Guitte^a^ | Middle-distant  Near-to-road | 93  186 | 768  970 |  | 40  80^a^ |
| Fishing villages:  Kaesai  Basara  Kouri (Topio) | Remote island  Remote island  Remote island | 70  69  47 | 315  286  140 |  | 30  30  20 |
| Pastoral villages:  Dandi  Ngurutu | Near-to-road/forests  Remote | 70  23 | 330  121 |  | 30  10 |
| Total | (558) (2930) (240) | | | | |
| Selection criteria^c^ | - Close proximity to the Lake waters - Presence of specific climate markers (e.g. droughts and water scarcity) - Existence of local governance structure (e.g. the presence of local chiefs or *bulamas*) - Dependence of local population on Lake Chad resources - Accessible by road, canoe or bush/forest paths - Willingness to participate in the study | | | | |
|  |  | | | | |
|  |  | | | | |

* Estimates are based on personal communications with local chiefs. Quota per village was based on 43% of the estimated households.

^a^ Near to road village is within vehicle access (120 – 130 km) from N’Djamena and have a central market and bus/fuel stations; the middle-distant village is a long way off the paved roads (about 150 km from N’Djamena), accessible through unmarked tracks by motor bikes; remote islands where the fishermen live are accessible by boat or canoe (they surround middle distant and near-to-road villages); vehicle or motor bike access to remote locations is difficult without a guide who is familiar with the rough terrain. Access is usually not possible during rainy seasons.

^b^ Guitte is a ‘mixed’ village where majority of households engage in either farming and herding activities or both; income-source ranking enabled the selection of 40 farmers and 40 herders from the village.

Note: quota scheme (number of households selected per village) is based on village sizes (i.e. 43% of estimated households per village).

References

Abdelali-Martini M, Hamza R (2014) How do migration remittances affect rural livelihoods in drylands? *Journal of International Development* 26: 454–470

Baird T, Gray C (2014) Livelihood diversification and shifting social networks of exchange: a social network transition? *World Development* 60:14–30

Bates B, Kundzewicz Z,Wu S, Palutikof J eds (2008) Climate change and water. Technical paper of the Intergovernmental Panel on Climate Change, IPCC Secretariat, Geneva 210 pp.

Butler C, Gates S (2012) African range wars: climate, conflict, and property rights. *Journal of Peace Research* 49 (1): 23–34

Codjoe S, Owusu G (2011) Climate change/variability and food systems: evidence from the Afram Plains, Ghana. *Regional Environmental Change* 11 (4): 753–765

Conca B K (2006) The new face of water conflict. *Navigating Peace Issue* 3, Wilson Center, USA

Freitas A (2013) Water as a stress factor in sub-Saharan Africa ­. European Union Institute of Security Studies Brief 12. [Online] URL: http://www.iss.europa.eu/uploads/media/Brief_12.pdf

Funder M, Bustamante R, Cossio V, Huong P, van Koppen B, Mweemba C, Nyambe I, Phuong L, Skielboe T (2012) Strategies of the poorest in local water conflict and cooperation -evidence from Vietnam, Bolivia and Zambia. *Water Alternatives* 5(1): 20–36

Geest K, Warner K (2015) Vulnerability, coping and loss and damage from climate events. In: Collins A (ed) Hazards, Risks, and Disasters in Society, pp 121 - 144. doi.org/10.1016/B978-0-12-396451-9.00008-1

Goulden M, Adger N, Allison E, Conway D (2013) Limits to resilience from livelihood diversification and social capital in lake social–ecological systems. *Annals of the Association of American Geographers* 103 (4): 906–924

Hahn M, Riederer A, Foster S (2009) The livelihood vulnerability index: a pragmatic approach to assessing risks from climate variability and change—A case study in Mozambique. *Global Environmental Change* 19 (1): 74–88

IPCC (2014) Summary for policymakers. In: Climate Change 2014: Impacts, Adaptation, and Vulnerability. Part A: Global and Sectoral Aspects. Contribution of Working Group II to the Fifth Assessment Report of the Intergovernmental Panel on Climate Change [Field, C.B., V.R. Barros, D.J. Dokken, K.J. Mach, M.D. Mastrandrea, T.E. Bilir, M. Chatterjee, K.L. Ebi, Y.O. Estrada, R.C. Genova, B. Girma, E.S. Kissel, A.N. Levy, S. MacCracken, P.R. Mastrandrea, and L.L. White (eds)]. Cambridge University Press, Cambridge, United Kingdom and New York, NY, USA, pp 1-32

Kotir J (2011) Climate change and variability in Sub-Saharan Africa: A review of current and future trends and impacts on agriculture and food security. *Environment, Development and Sustainability* 13 (3): 587–605

Ludwig R, Zografos C, Kallis G (2011) Towards an inter-disciplinary research agenda on climate change, water and security in Southern Europe and neighboring countries. *Environmental Science and Policy* 14 (7):794–803

Mubaya C, Njuki J, Mutsvangwa E, Mugabe F, Nanja D (2012) Climate variability and change or multiple stressors? Farmer perceptions regarding threats to livelihoods in Zimbabwe and Zambia. *Journal of environmental management* 102: 9–17

Nunan F (2010) Mobility and fisherfolk livelihoods on Lake Victoria: Implications for vulnerability and risk. *Geoforum* 41 (5): 776–785

Onuoha F (2009) Environmental degradation, livelihood and conflicts: a focus on the implications of the diminishing water resources of Lake Chad in North-Eastern Nigeria. *African Journal on Conflict Resolution* 8 (2): 35–61

Paavola J (2008) Livelihoods, vulnerability and adaptation to climate change in Morogoro, Tanzania. *Environmental Science and Policy* 11 (7): 642–654

Rast W (2014) The 15th World Lake Conference: an overview of an informative event. *Lakes and Reservoirs: Research and Management* 19 (4): 237–239

Sallu S, Twyman C, Stringer L (2010) Resilient or vulnerable livelihoods? Assessing livelihood dynamics and trajectories in rural Botswana. *Ecology and Society* 15 (4): 3

Sarr B (2012) Present and future climate change in the semi-arid region of West Africa: A crucial input for practical adaptation in agriculture. *Atmospheric Science Letters* 13 (2): 108–112

Scoones I (2009) Livelihoods perspectives and rural development. *Journal of Peasant Studies* 36 (1): 171–196

Shah K, Dulal H, Johnson C, Baptiste A (2013) Understanding livelihood vulnerability to climate change: applying the livelihood vulnerability index in Trinidad and Tobago. *Geoforum* 47: 125–137

De Souza R (2015) Demographic resilience: linking population dynamics, the environment, and security. *SAIS Review of International Affairs* 35 (1): 17–27.

Tambo J, Abdoulaye T (2013) Smallholder farmers’ perceptions of and adaptations to climate change in the Nigerian savanna. *Regional Environmental Change* 13 (2): 375–388.
